# Altered mismatch response of inferior parietal lobule in amnestic mild cognitive impairment: A magnetoencephalographic study

**DOI:** 10.1111/cns.13691

**Published:** 2021-08-04

**Authors:** Pin‐Yu Chen, Hui‐Yun Hsu, Yi‐Ping Chao, Rui Nouchi, Pei‐Ning Wang, Chia‐Hsiung Cheng

**Affiliations:** ^1^ Department of Occupational Therapy and Graduate Institute of Behavioral Sciences Chang Gung University Taoyuan Taiwan; ^2^ Laboratory of Brain Imaging and Neural Dynamics (BIND Lab) Chang Gung University Taoyuan Taiwan; ^3^ Graduate Institute of Biomedical Engineering Chang Gung University Taoyuan Taiwan; ^4^ Department of Neurology Chang Gung Memorial Hospital Linkou Taiwan; ^5^ Institute of Development, Aging and Cancer (IDAC) Tohoku University Sendai Japan; ^6^ Smart Aging Research Center (S.A.R.C) Tohoku University Sendai Japan; ^7^ Division of General Neurology Department of Neurological Institute Taipei Veterans General Hospital Taipei Taiwan; ^8^ Department of Neurology National Yang Ming Chiao Tung University Taipei Taiwan; ^9^ Healthy Aging Research Center Chang Gung University Taoyuan Taiwan; ^10^ Department of Psychiatry Chang Gung Memorial Hospital Linkou Taiwan

**Keywords:** mismatch negativity (MMN), amnestic mild cognitive impairment (aMCI), inferior parietal lobule (IPL), magnetoencephalography (MEG)

## Abstract

**Background:**

Mismatch negativity (MMN) reflects the functional integrity of sensory memory function. With the advantages of independence of individual's focused attention and behavioral cooperation, this neurophysiological signal is particularly suitable for investigating elderly with cognitive decline such as amnestic mild cognitive impairment (aMCI). However, the existing results remain substantially inconsistent whether these patients show deficits of MMN. In order to reconcile the previous disputes, the present study used magnetoencephalography combined with distributed source imaging methods to determine the source‐level magnetic mismatch negativity (MMNm) in aMCI.

**Methods:**

A total of 26 healthy controls (HC) and 26 patients with aMCI underwent an auditory oddball paradigm during the MEG recordings. MMNm amplitudes and latencies in the bilateral superior temporal gyrus, inferior frontal gyrus, and inferior parietal lobule (IPL) were compared between HC and aMCI groups. The correlations of MMNm responses with performance of auditory/verbal memory tests were examined. Finally, MMNm and its combination with verbal/auditory memory tests were submitted to receiver operating characteristic (ROC) curve analysis.

**Results:**

Compared to HC, patients with aMCI showed significantly delayed MMNm latencies in the IPL. Among the patients with aMCI, longer MMNm latencies of left IPL were associated with lower scores of Chinese Version Verbal Learning Test (CVVLT). The ROC curve analysis revealed that the combination of MMNm latencies of left IPL and CVVLT scores yielded a moderate accuracy in the discrimination of aMCI from HC at an individual level.

**Conclusions:**

Our data suggest dysfunctional MMNm in patients with aMCI, particularly in the IPL.

## Graphical abstract

This study uses MEG to compare source‐level magnetic mismatch negativity (MMNm) in healthy elderly and patients with amnestic mild cognitive impairment (aMCI). Patients with aMCI show MMNm prolongation in the inferior parietal lobule, and such a deficit correlates with poorer verbal learning test.

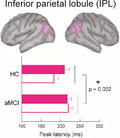

## 1 INTRODUCTION

Mild cognitive impairment (MCI) has been proposed to be an intermediate stage of cognitive dysfunction between normal aging and dementia.^1^ Unlike patients with dementia, those with MCI still maintain their independence in most of activities of daily living (ADL) with minimal aids or assistance; however, cognitive deficits are noticeable and detected by neuropsychological assessments in these patients.^2^, ^3^, ^4^ The concept of MCI is further categorized as amnestic MCI (aMCI) and non‐amnestic MCI (naMCI).^2^, ^5^ Clinically, aMCI is characterized as apparent deficits in memory rather than other cognitive functions and is shown to be strongly associated with the development of Alzheimer's disease (AD), the most common type of dementia.^6^, ^7^ Since AD would cause severe ADL dysfunction which in turn increases the health care and economic burdens, the early and accurate diagnosis of aMCI prior to AD is imperative.

Up to the date, the diagnosis of aMCI is based on the clinical criteria.^5^, ^6^, ^7^ Although some biomarkers, such as beta amyloid or tau levels of cerebrospinal fluid (CSF) and positron emission tomography (PET) imaging with C‐labeled Pittsburgh compound‐B, have been proposed for their utility in the diagnosis of aMCI due to AD, the existing data do not provide strong evidence for the routine use of these biomarkers in clinical practice.^8^, ^9^, ^10^ Furthermore, with the fact that PET imaging is expensive and CSF is not easy to collect from each patient with aMCI, the search for other markers is needed. Neurophysiological recordings using electroencephalography (EEG) or magnetoencephalography (MEG) have been demonstrated as promising tools in the AD research^11^, ^12^, ^13^ and can also be considered suitable for the studies of aMCI. When applying neurophysiological recordings in the studies of neurodegenerative diseases, a task or a signal that is largely independent of the individual's focused attention, motivation, or behavioral cooperation is a key element for its clinical utility and future application. Mismatch negativity (MMN), or its magnetic counterpart (MMNm), is one of the neurophysiological signals that possess these advantages.^14^, ^15^, ^16^, ^17^

MMN/MMNm is an automatic cortical activity elicited by a passive oddball task, in which a sequence of identical auditory stimuli (standards) is occasionally interrupted by deviant sounds (deviants) differing in any of perceptual characteristics, such as pitch, duration, intensity, or location.^16^, ^17^, ^18^, ^19^ The generation of the MMN/MMNm has been interpreted as a pre‐attentive cognitive process indexing functional integrity of sensory memory and accuracy in detecting changes.^20^, ^21^, ^22^ Although the existing literature has shown the deficits of MMN/MMNm in patients with AD,^23^, ^24^, ^25^, ^26^ the results in aMCI are substantially inconsistent. There are only five studies so far investigating the auditory MMN in patients with aMCI/MCI.^27^, ^28^, ^29^, ^30^, ^31^ Mowszowski et al. used tone duration as deviants and found that compared to healthy controls (HC), patients with MCI exhibited reduced MMN amplitudes.^30^ Similar finding of reduced MMN amplitudes in patients with aMCI was also reported by Lindin and colleagues, who used tone frequency as deviants.^29^ However, Ji and colleagues, using frequency deviants as well, found defects of MMN latencies, rather than MMN amplitudes, in patients with aMCI as compared with HC.^28^ In contrast to aforementioned three studies, two studies reported no declined or even improved MMN responses in patients with MCI as compared with HC.^27^, ^31^ As a whole, it is difficult to draw a precise conclusion regarding the MMN in aMCI/MCI.

One of the major causes leading to extremely controversial results is attributed to the mixture of patient characteristics. For example, Mowszowski et al. and Tsolaki et al. recruited both aMCI and naMCI in their studies.^30^, ^31^ The second one is related to the different task instructions among the studies. MMN is conventionally obtained through a passive oddball paradigm, while Tsolaki et al. used an active oddball task in which the subjects were asked to respond to infrequent targets.^31^ Finally, all the existing studies investigated MMN by means of EEG, whose signals are potentially distorted by different tissues (e.g., brain, CSF, and skull). Also, due to the limited spatial resolution, the brain activities recorded from the scalp electrodes provide little information of the source activation of MMN. Therefore, in order to address these caveats, the present study aimed to use a whole‐head MEG, which has a spatial resolution with a millimeter scale and a temporal resolution with a millisecond scale, to examine the spatiotemporal dynamics of MMNm activities in patients with aMCI.

The specific goals of this study were three‐fold. Firstly, based on the selected regions of interest (ROIs), we attempted to determine whether, at a group level, MMNm amplitudes and latencies at the cortical level would be reduced in the patients with aMCI versus HC group. Secondly, we sought to examine whether the MMNm in the ROIs, which exhibited significant between‐group differences (if detected), would show significant associations with cognitive performance related to auditory/verbal memory tests, including Chinese Version Verbal Learning Test, Logical Memory A of the Wechsler Memory Scale, and Digit Span Backward. Finally, we used the MMNm activity and its combination with auditory/verbal memory tests to examine the discrimination accuracy between HC and aMCI at an individual level.

## 2 METHODS

### 2.1 Participants

This study included 26 patients with aMCI (11 males, mean age = 71.96 ± 1.88 years) from the memory clinics at Taipei Veterans General Hospital. Clinical diagnosis of aMCI was made by the neurologist (PNW) based on clinical criteria.^32^ The patients had episodic memory impairment (Chinese Version Verbal Learning Test [CVVLT] below 1.5 standard deviation of the norm data) along with normal functioning in ADL. MRI and laboratory examinations were used to rule out stroke, severe white matter diseases, and tumors. In addition, their scores of Mini‐Mental State Examination (MMSE) were ≧24 and without dementia.^33^ A total of 26 community‐dwelling older adults (9 males, mean age = 65.81 ± 1.45 years) were recruited as the HC group. All the subjects had no history of major psychiatric disorders, alcoholism, epilepsy, polypharmacy, or other systematic diseases that potentially have detrimental effects on cognitive function. They also self‐reported no hearing impairment, and normal or corrected‐to‐normal vision.

The Institutional Review Board of Taipei Veterans General Hospital (Taipei, Taiwan) approved this research project. Written informed consent was obtained from each subject after a complete explanation of the study procedure.

### 2.2 Neuropsychological assessments

Each subject was evaluated by a standardized battery of neuropsychological assessments, including MMSE, CVVLT, Logic Memory (LM) A of the Wechsler Memory Scale, Rey‐Osterrieth Complex Figure Test, Verbal Fluency Test, Boston Naming Test, Digit Span Forward, Digit Span Backward (DSB), and Trail Making Test (Table 1).

**TABLE 1:** Demographic data and neuropsychological measures (mean ± SEM)

	HC (*n* = 26)	aMCI (*n* = 26)	*p* values
Sex (male/female)	9/17	11/15	0.10
Age	65.81 ± 1.45	71.96 ± 1.88	0.10
Race	Chinese	Chinese	—
Medication	1 BZD	3 BZD 2 SSRIs	—
MMSE	29.04 ± 0.20	28.35 ± 0.24	0.03*
CVVLT_Total	30.92 ± 0.66	25.65 ± 0.81	<0.001*
CVVLT_Delayed	8.27 ± 0.19	6.35 ± 0.30	<0.001*
LM_Immediate	15.27 ± 0.62	10.58 ± 0.75	<0.001*
LM_Delayed	14.19 ± 0.73	8.23 ± 0.78	<0.001*
CFT_Copy	32.8 ± 0.50	31.54 ± 0.66	0.13
CFT_Immediate	24.77 ± 1.23	19.10 ± 1.48	0.01*
CFT_Delayed	24.35 ± 1.32	17.73 ± 1.43	0.001*
Verbal fluency	18.89 ± 0.88	15.46 ± 0.97	0.01*
BNT_Spontaneous	26.69 ± 0.49	26.88 ± 0.52	0.80
BNT_Semantic cued	0.50 ± 0.15	0.23 ± 0.10	0.14
BNT_Phonemic cued	1.85 ± 0.27	1.46 ± 0.32	0.36
Digit Span Forward	8.31 ± 0.23	8.04 ± 0.22	0.40
Digit Span Backward	5.73 ± 0.28	4.62 ± 0.25	0.01*
Trail Making Test A (sec)	18.54 ± 3.29	13.50 ± 0.75	0.24
Trail Making Test B (sec)	36.0 ± 5.23	47.62 ± 5.35	0.13

Abbreviations: aMCI, amnestic mild cognitive impairment; BNT, Boston Naming Test; BZD, benzodiazepines; CFT, Rey‐Osterrieth Complex Figure Test; CVVLT, Chinese Version Verbal Learning Test; HC, healthy control; LM, Logical Memory A of the Wechsler Memory Scale; MMSE, Mini‐Mental State Examination; SEM, standard error of the mean; SSRIs, selective serotonin reuptake inhibitors.

* *p* < 0.05.

Based on that the MMNm reflects functional integrity of auditory sensory memory and the patients with aMCI exhibit impairments in memory function, we specifically examined the following auditory/verbal memory tests in relation to MMNm:

1. CVVLT: Nine two‐character nouns were spoken to the subject to measure total (learning over 4 trials) and delayed recall scores.^34^
2. LM: a brief story was spoken to the subject to measure immediate and delayed recall memory function.^35^
3. DSB: a series of digits were spoken to the subject to measure the auditory working memory.^36^

### 2.3 MEG recordings

The subjects were presented with an auditory oddball paradigm consisting of 85% standard stimuli (1000 Hz, 70 dB, 100 ms) and 15% deviant stimuli (900 Hz, 70 dB, 100 ms) in a pseudo‐random order in which two deviants were separated by at least one standard. The interstimulus interval was 1000 ms. During the whole experiment, the subjects were asked to watch a silent movie with subtitles and ignore the auditory stimuli.

The neuromagnetic responses to standards and deviants were recorded by a whole‐head 306‐channel MEG system (Elekta‐Neuromag), consisting of 102 magnetometers and 204 gradiometer. The online sampling rate and bandpass filter were set at 1000 Hz and 0.1–200 Hz, respectively. In addition to three anatomical landmarks and four head position indicators, further ~100 head points were uniformly digitized on the head surface with a 3D digitizer. Electrooculography (EOG) and electrocardiography (ECG) were used to monitor the eye blinks and cardiac artifacts. The mean numbers of deviants did not significantly differ between HC (121.73 ± 3.84) and aMCI (116.04 ± 2.93) groups (*p* = 0.245).

### 2.4 MEG data analysis

The MEG raw data were pre‐processed by MaxFilter to suppress external and internal interference^37^, ^38^ and by signal space projections (SSP) to correct the trials contaminated by eye and cardiac artifacts.^39^, ^40^ The artifact‐free standards and deviants were separately epoched into 500 ms, including a 100‐ms pre‐stimulus baseline. The offline bandpass filter was set at 0.1–30 Hz. In an attempt to achieve an equivalent signal‐to‐noise between standards and deviants, the number of standards was randomly selected to match the number of deviants. The MMNm was measured as a difference waveform obtained by subtracting neuromagnetic responses to standards from those to deviants.^16^, ^17^, ^22^ The time window of MMNm was defined as 150–300 ms after the stimulus onset.

An overlapping‐sphere method was used to resolve the forward problem. The cortically constrained source imaging was subsequently performed by means of depth‐weighted minimum norm estimate (MNE) over a set of ~15,000 dipoles distributed on the cortex.^41^ The individual's MNE map was then rescaled to fit the previously defined head points, with the default parameter settings in the brainstorm software.^42^ The MNE maps of MMNm from each group were averaged onto the ICBM152 brain template for further analysis.

The existing literature and our previous works suggest that neural generators of MMNm include superior temporal gyrus (STG),^16^, ^17^ inferior frontal gyrus (IFG),^43^, ^44^ and inferior parietal lobule (IPL).^45^, ^46^ Therefore, we identified 6 ROIs on the Desikan‐Killiany cortical atlas installed in the Brainstorm software: (1) left STG (SCS coordinate = [15, 59, 40], vertice number = 290, area = 47.81 cm^2^); (2) right STG (SCS coordinate = [23, −59, 37], vertice number = 257, area = 43.98 cm^2^); (3) left IFG (SCS coordinate = [40, 56, 60], vertice number = 139, area = 20.90 cm^2^); (4) right IFG (SCS coordinate = [43, −57, 62], vertice number = 118, area = 17.05 cm^2^); (5) left IPL (SCS coordinate = [−42, 50, 79], vertice number = 351, area = 52.71 cm^2^); and (6) right IPL (SCS coordinate = [−36, −50, 82], vertice number = 421, area = 61.33 cm^2^). The ROI identification on the Desikan‐Killiany brain template has been reported in other studies.^47^, ^48^

The MNE magnitude of each dipole (i.e., vertice) was normalized in relation to its fluctuations over 100‐ms, yielding a *z*‐score map. The peak amplitudes (*z*‐score) and peak latencies (ms) of MMNm were derived from each ROI.

### 2.5 Statistical analysis

All the data were presented as mean ± one standard error of the mean (SEM), and *p* values less than 0.05 were considered as statistically significant. The numerical data were normally distributed as verified by the Kolmogorov‐Smirnov tests (*Z* < 0.999, *p* > 0.271). Between‐group differences of demographic and neuropsychological data were compared using independent two‐sample *t*‐tests or chi‐square tests as appropriate. Two‐way mixed ANOVAs, with group (HC and aMCI) as a between‐subject factor and hemisphere (left and right) as a within‐subject factor, were applied to compare MMNm amplitudes and latencies in each identified ROI. Furthermore, age and gender were entered as covariate variables. Greenhouse‐Geisser epsilon correction was applied when the assumption of sphericity was violated. Partial eta squared was used to estimate the effect size.

Based on the ROIs with significant between‐group differences, partial correlations, with age and gender as covariates, were used to determine the associations between MMNm responses and scores of auditory/verbal memory tests (i.e., CVVLT_Total, CVVLT_Delayed, LM_Immediate, LM_Delayed, DSB) among the patients with aMCI. The significance was further adjusted for multiple comparisons by means of Bonferroni approach.

Finally, based on the significant between‐group differences of MMNm responses and significant correlational data, MMNm and its combination with verbal/auditory memory tests were submitted to receiver operating characteristic (ROC) curve analysis. For the area under the curve (AUC), an AUC between 0.5 and 0.7 was considered less accurate, an AUC between 0.7 and 0.9 was considered moderately accurate, and an AUC above 0.9 was considered very accurate.^49^

## 3 RESULTS

**FIGURE 1:**
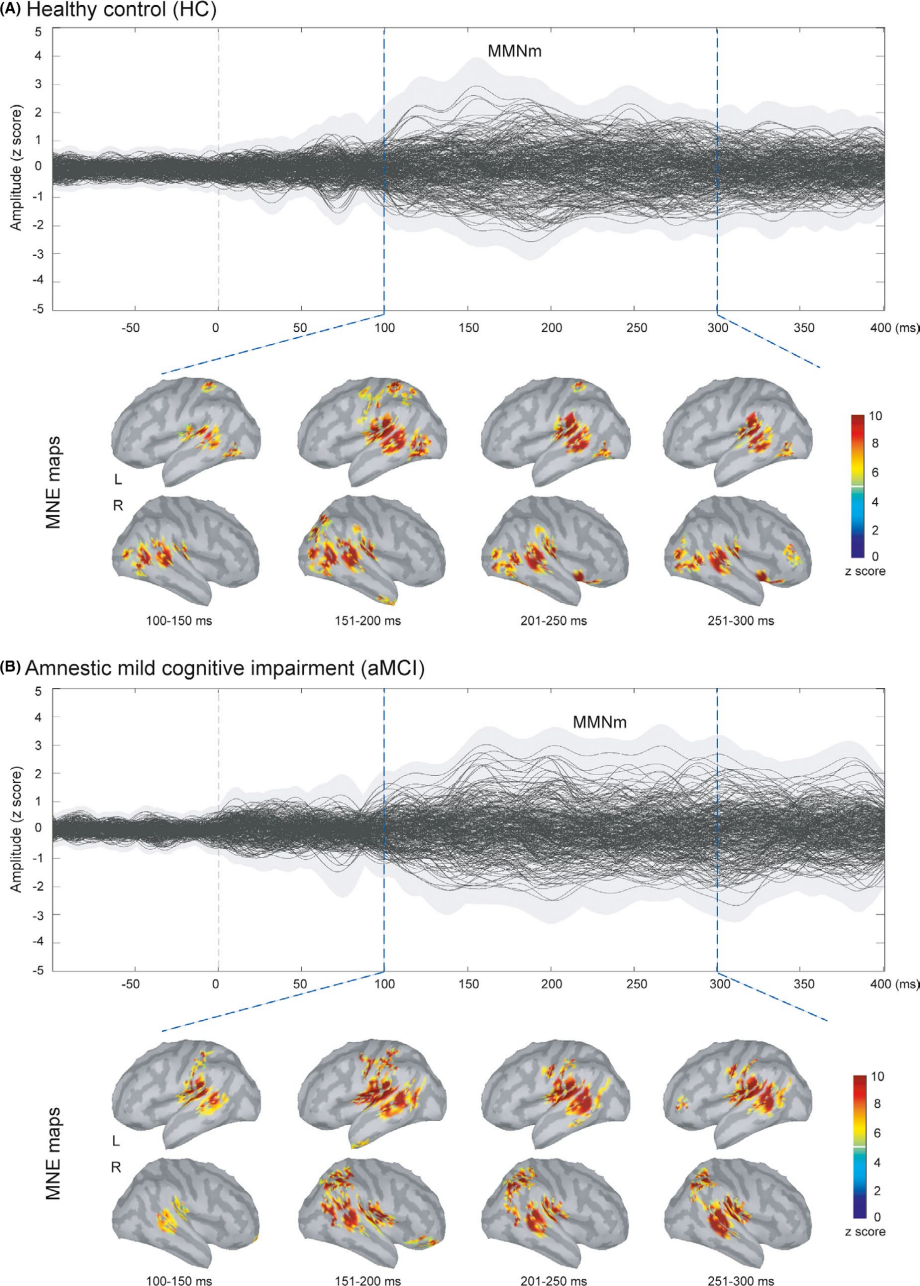
Magnetic mismatch negativity (MMNm) is the difference in neuromagnetic responses to standard and deviant auditory stimuli. The grand‐averaged butterfly plots of the sensor waveforms are illustrated for healthy control (HC, *n* = 26) and amnestic mild cognitive impairment (aMCI, *n* = 26) groups. The spatiotemporal dynamics of the MMNm reconstructed by the depth‐weighted minimum norm estimate (MNE) were obtained via the average of cortical responses across every 50‐ms time frame from 100 to 300 ms

**FIGURE 2:**
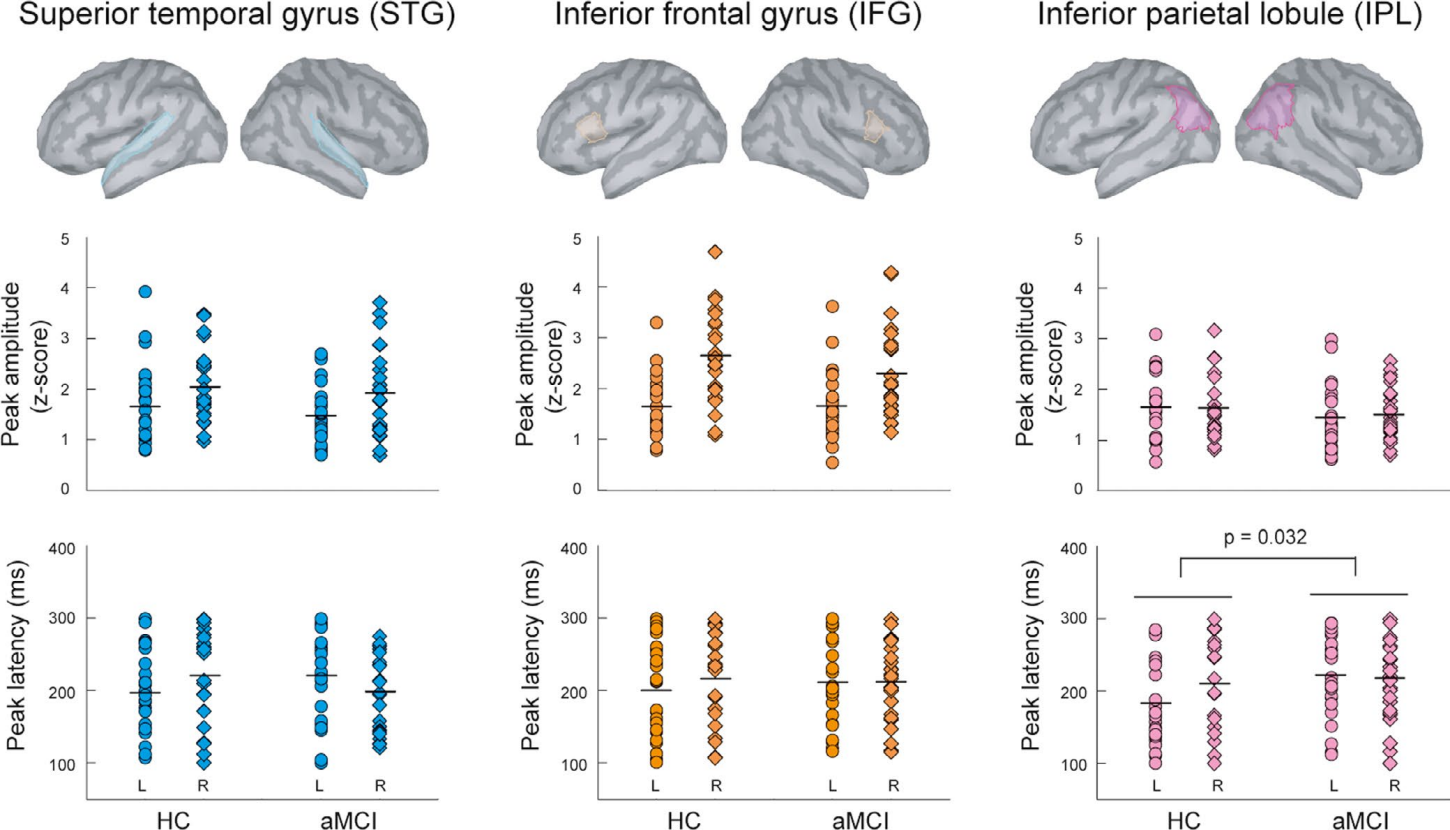
Six regions of interest related to the MMNm were identified in the bilateral superior temporal gyrus (STG), inferior frontal gyrus (IFG), and inferior parietal lobule (IPL). Results of two‐way mixed ANOVAs, with age and gender as covariates, showed that compared to healthy controls (HC), patients with amnestic mild cognitive impairment (aMCI) exhibited delayed MMNm latencies in the IPL. The horizontal bars in each dot plot represent the mean values

Figure 1 displays the grand‐averaged sensor waveforms of MMNm in HC (*n* = 26) and aMCI (*n* = 26) groups. The results of MNE source reconstruction reveal cortical activation of temporal, frontal, and parietal cortices between 100 and 300 ms. Based on the identified ROIs, we performed the two‐way mixed ANOVAs to investigate the between‐group differences of MMNm amplitudes and latencies (Figure 2). Compared to HC, patients with aMCI demonstrated significant prolongation of MMNm latencies in the left IPL (*F* = 4.893, *p* = 0.032, two‐tailed, partial eta squared = 0.093).

**FIGURE 3:**
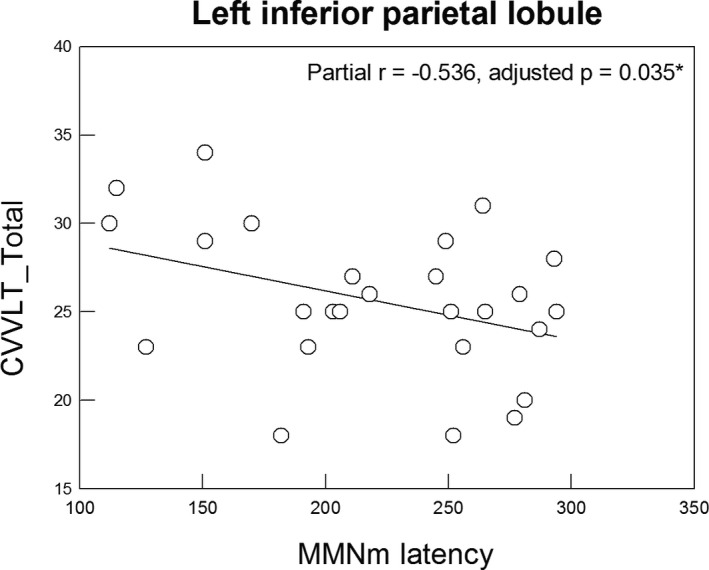
Significant association between MMNm latencies of the left inferior parietal lobule and total scores of Chinese Version Verbal Learning Test (CVVLT_Total) among the patients with amnestic mild cognitive impairment was found (*p* value was adjusted by Bonferroni approach)

Since significant between‐group differences of MMNm latencies were detected in the IPL, it was interesting to further examine whether MMNm latencies in the left and right IPL were associated with the performance of auditory/verbal memory as assessed by CVVLT_Total, CVVLT_Delayed, LM_Immediate, LM_Delayed, and DSB. The correlational results showed that in the left IPL (Figure 3), delayed MMNm latencies were significantly associated with lower scores of CVVLT_Total (partial *r* = −0.536, adjusted *p* = 0.035, two‐tailed) among the patients with aMCI. No other significant results were found after the Bonferroni corrections (CVVLT_Delayed: *r* = −0.422, adjusted *p* = 0.20; LM_Immediate: *r* = −0.377, adjusted *p* = 0.345; LM_Delayed: *r* = −0.143, adjusted *p* = 1.0; DSB: *r* = −0.081, adjusted *p* = 1.0).

**FIGURE 4:**
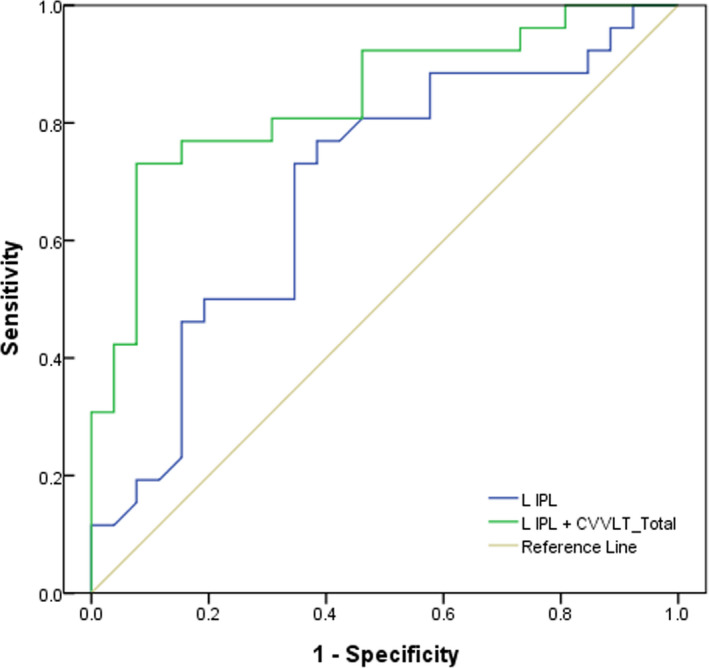
Receiver operator characteristic (ROC) curve analysis of MMNm latency of the left inferior parietal lobule (IPL) and its combination with total scores of Chinese Version Verbal Learning Test (CVVLT_Total)

Given that MMNm latencies of the left IPL and CVVLT_Total showed the most significant results at a group level, we further applied the ROC curve analysis to determine whether the MMNm latency of left IPL or its combination with CVVLT_Total could discriminate aMCI patients from HC at an individual level. The AUC of MMNm latencies of the left IPL was 0.689 (sensitivity = 76.9%, specificity = 61.5%, *p* = 0.020), considered less accurate (Figure 4). It was notable that the MMNm latencies of the left IPL together with CVVLT_Total reached a moderate accuracy in the discrimination aMCI from HC (AUC = 0.842, sensitivity = 80.8%, specificity = 69.2%, *p* < 0.001).

## 4 DISCUSSION

The present study used MEG recordings and MNE imaging methods to compared the source‐level MMNm responses between HC and aMCI groups. Our results yielded three major findings. Firstly, compared to HC, patients with aMCI exhibited delayed MMNm latency in the IPL. Secondly, MMNm latencies of the left IPL were significantly associated with the performance of memory function measured by Chinese Version Verbal Learning Test. Finally, the ROC curve analysis showed that combining MMNm latencies of the left IPL and CVVLT could accurately discriminate aMCI from HC at an individual level.

Up to the date, the existing 5 MMN studies in aMCI/MCI all applied EEG recordings and analyzed MMN activities on the surface electrodes. Unfortunately, the results were quite inconsistent. Our present MEG study analyzed tone‐elicited MMNm activities at a cortical level revealed that MMNm latencies were significantly delayed in the IPL, in consistent with the previous studies.^28^ However, unlike other studies showing attenuated MMNm amplitudes in aMCI/MCI,^29^, ^30^ we did not find significant between‐group differences of MMNm amplitudes in any of identified ROIs. One plausible reason to account for this discrepancy was that patients with aMCI and naMCI were mixed in some of the studies.^30^, ^31^ Another interpretation was related to different task design (e.g., tone deviants vs. novelty deviants, passive oddball vs. active oddball). For example, Lindin and colleagues reported a reduced MMN amplitude in patients with aMCI versus HC, and such a deficit was attributed to novelty deviants rather than tone deviants.^29^ Similarly, another study showed that the novelty‐elicited MMN was found to be shortened in the patients with aMCI; the tone‐elicited MMN amplitudes or latencies did not significantly differ between HC and aMCI groups.^27^ In terms of task instruction, all the studies used passive oddball paradigm^27^, ^28^, ^29^, ^30^ except for that Tsolaki and colleagues carried out an active task in which the subjects were asked to make a response to target tones.^31^

Notably, we found that MMNm latencies of the left IPL were significantly correlated with the performance of CVVLT. Previous studies have reported significant associations of MMN responses with attention,^50^ working memory,^51^ planning,^52^ and verbal learning and memory^52^ in the healthy adults. Here, we further indicated that more delayed MMNm latencies, particularly in the left IPL, were associated with worse performance of verbal learning tests among the patients with aMCI. Mounting evidence from structural and functional studies has shown that the left inferior parietal area is related to auditory working memory and episodic memory.^53^, ^54^ For example, lesion studies have shown that the left IPL is a key region for the storage of verbal short‐term memory.^55^, ^56^ Compared to HC, gray matter atrophy of the IPL has been well documented in patients with aMCI.^57^, ^58^, ^59^ It should be noted that the one of the promising diagnostic values for distinguishing aMCI from HC was the gray matter volume of left IPL (left supramarginal gyrus in their article).^58^ Other studies by means of neuromodulation technique further provided empirical evidence to shed lights on the role of left IPL in the processing of verbal short‐term storage and recall. For example, it has been shown that repetitive transcranial magnetic stimulation (rTMS) on left IPL disrupted the subjects' performance on episodic memory retrieval.^60^ Similarly, rTMS on the left or right IPL (i.e., close to the intraparietal sulcus in their article) led to significant deterioration of performance in verbal working memory.^61^ Taken together, our current data extended the previous knowledge to reveal that patients with aMCI showed an apparent deficit of pre‐attentive change detection in the left IPL, and such a deficit furtherer had a detrimental influence on the verbal learning test.

Our ROC curve analysis revealed that MMNm latency of the left IPL was not an acceptable indicator for the discrimination of aMCI from HC (AUC = 0.769). However, the combination of MMNm and CVVLT largely improved the diagnostic power (AUC = 0.842, sensitivity = 80.8%, specificity = 69.2%) to achieve a moderate accuracy. A previous study using a visual oddball task has shown that P300 latency could serve as an excellent indicator for the discrimination between HC and MCI (AUC = 0.97), with the sensitivity of 80% and specificity of 100%.^62^ Despite such a high accuracy, it should be noted that P300 should be obtained through the cooperation of the subjects including sustained attention and motivation, which were usually poor in the patients with neurodegenerative disorders. From the clinical perspective, a pre‐attentive/automatic indicator with a satisfactory accuracy is much more expected since it can not only suit for the cross‐investigation but also for the monitoring of the disease progression such as from aMCI to AD. Thus, it merits further research using pre‐attentive signals,^48^, ^63^ resting‐state brain activities,^64^, ^65^ or the combination of these parameters to perfectly separate the aMCI from HC at an individual level.

The present study had several limitations. Firstly, the paradigm we used here was traditional version without a precise control for the physical properties of the auditory stimuli. For example, the different magnitudes of refractoriness between standards (85%) and deviants (15%) might cause the non‐memory‐based N100/N100m to contaminated the memory‐based MMN/MMNm.^66^, ^67^, ^68^ Over the past decades, a controlled block along with the oddball paradigm has been designed to successfully address this issue. However, in terms of future clinical application, we considered that the traditional oddball paradigm would be easier to operate and the recording time would be short. Secondly, we could not entirely preclude the effects of medicine (e.g., benzodiazepines) on the MMNm responses though these patients were instructed to refrain from taking their medication 24 h prior to MEG recordings. Finally, we did not carefully control the hearing threshold from each subject. However, it has been evident that there was no age‐related difference of hearing threshold at the frequency of 1000 Hz.^69^ Considering that the tone frequencies we used in the present study were 1000 and 900 Hz, we reasoned that all the participants could successfully encode the auditory stimuli for the basic processing. Furthermore, since both of the HC and aMCI groups were composed of older adults and there was no significant difference of age, thus our sample was considered homogeneous in terms of auditory acuity.

In conclusion, we found that patients with aMCI showed MMNm prolongation selectively in the IPL compared with HC, and such aberration was associated with the performance of auditory/verbal memory (i.e., CVVLT). Our results also indicated that the combination of MMNm latencies of left IPL with CVVLT could adequately discriminate patients with aMCI from HC at an individual level.

## CONFLICT OF INTEREST

The authors declare that they have no conflict of interest.

## AUTHOR CONTRIBUTIONS

CHC and PNW conceived and designed the work and acquired the data. PYC, HYH, YPC, and CHC analyzed the data. PYC, HYH, YPC, RN, and PNW participated in the discussion and provided the comments. PYC, HYH, and CHC wrote the paper. All of the authors have read and approved the manuscript.

## Data Availability

Data available on request from the authors.
